# Supplementary material for: The interactome of intact mitochondria by cross-linking mass spectrometry provides evidence for coexisting respiratory supercomplexes
Source: Mol Cell Proteomics. 2017 Dec 8;17(2):216–32. doi: 10.1074/mcp.RA117.000470 (PMC5795388; doi:10.1074/mcp.RA117.000470)
Supplement: Supplemental Data [file supp_RA117.000470_133922_0_supp_23978_fzffwf.zip › spectra_annotation/mito_spectra_annotation/337-1-14-1-3-1.pdf]

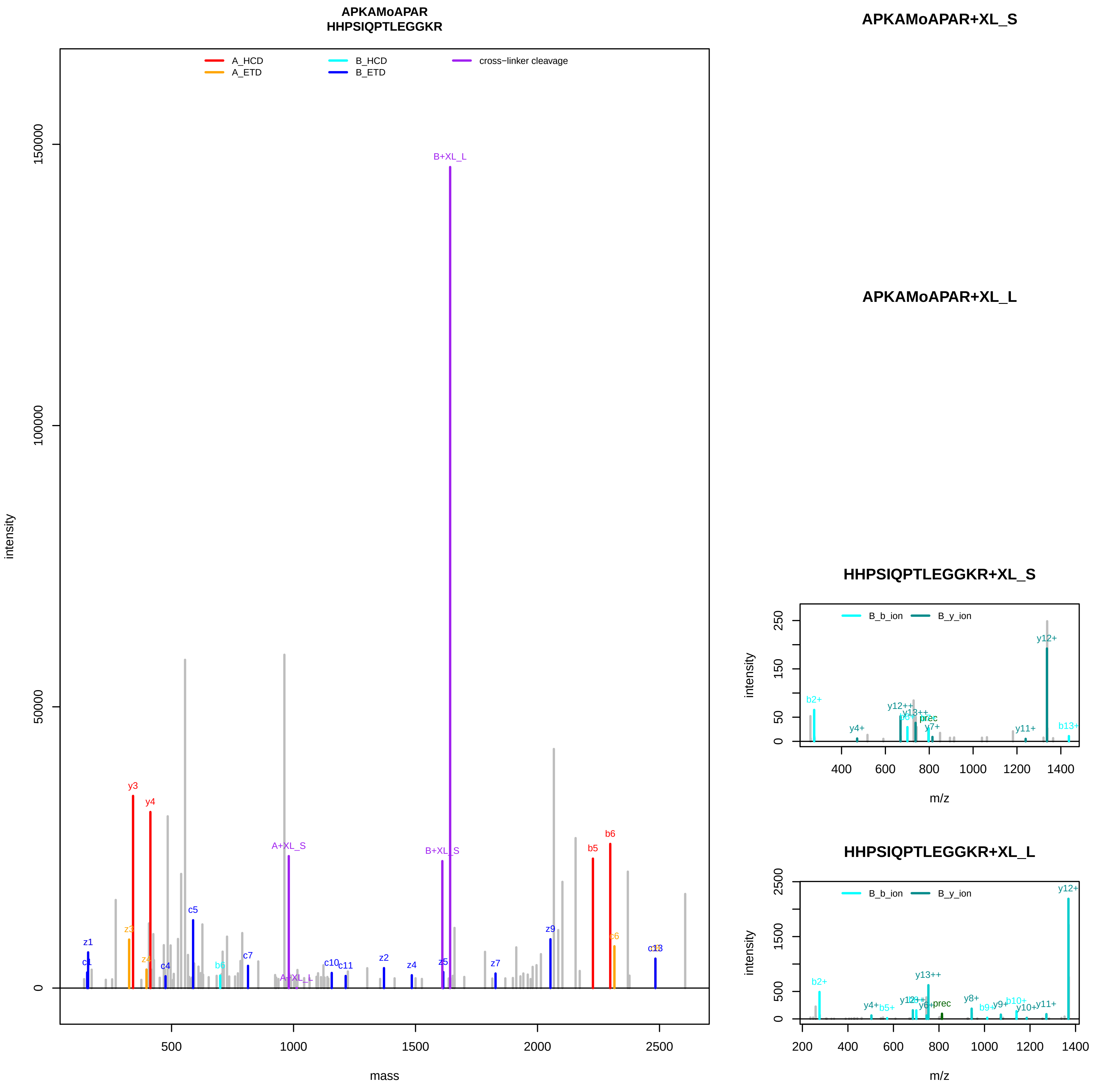

APKAMoAPAR
HHPSIQPTLEGGKR
A_HCD
A_ETD
B_HCD
B_ETD
cross−linker cleavage
intensity
mass
B+XL_L
A+XL_S
B+XL_S
A+XL_L
y3
y4
b5
b6
c1
z1
z3
z4
c4
c5
b6
c7
c10
c11
z2
z4
z5
z7
z9
c6
c13
APKAMoAPAR+XL_S
APKAMoAPAR+XL_L
HHPSIQPTLEGGKR+XL_S
HHPSIQPTLEGGKR+XL_L
B_b_ion
B_y_ion
m/z
b2+
y4+
y12++
y13++
prec
y7+
y11+
y12+
b13+
b5+
y6+
y8+
b9+
y9+
b10+
y10+
